# The outcome of neoadjuvant chemotherapy and the current trend of surgical treatment in young women with breast cancer: A multicenter real-world study (CSBrS-012)

**DOI:** 10.3389/fpubh.2023.1100421

**Published:** 2023-02-21

**Authors:** Yijun Li, Heyan Chen, Jianjun He, Zhimin Fan, Huimin Zhang

**Affiliations:** ^1^Department of Breast Surgery, The First Affiliated Hospital of Xi'an Jiaotong University, Xi'an, Shaanxi, China; ^2^Department of Breast Surgery, The First Hospital of Jilin University, Changchun, Jilin, China

**Keywords:** breast neoplasms, retrospective studies, neoadjuvant therapy, mastectomy, female

## Abstract

**Background and objectives:**

The relationship between age and the outcome of breast cancer neoadjuvant chemotherapy (NAC) remains controversial, and little is known about the choice of surgical treatment for young patients. In this multicenter real-world study, we sought to analyze the outcome of NAC as well as the current status and trend of surgical decision-making after NAC in young breast cancer patients.

**Methods:**

The medical records of patients from 20 hospitals in different regions of China were collected retrospectively. The study population included females diagnosed with cT1-4N0-3M0 breast cancer who received NAC from January 2010 to December 2020.

**Results:**

A total of 9,643 eligible patients were included, 1,945 (20.2%) of whom were ≤40 years old. Young patients tend to have a higher tumor stage and a higher proportion of Luminal B and triple-negative breast cancer (TNBC) tumors compared with the >40-year-old group. The breast pathological complete response (pCR) rate in the young group was 20.3%, and Luminal B tumor was more likely to obtain pCR in young patients. The implementation rate of breast-conserving surgery (BCS) and breast reconstruction surgery was higher in young patients and tended to increase over time. In different regions of China, there were great differences in the choice of surgical treatment after NAC among young patients.

**Conclusion:**

Breast cancer in young women has unique clinical characteristics, but age does not affect the overall pCR rate. In China, the BCS rate after NAC is increasing over time but is still at a low level.

## 1. Introduction

The diagnosis and treatment of breast cancer (BC) in young patients is a global problem. According to BC statistics in 2022, the incidence rate of BC in young individuals is increasing yearly. From 2015 to 2019, 5% of BC patients in the United States were younger than 40 years old at the time of diagnosis. In 2020, there were 10,850 new cases of young BC in the United States (1). In East Asia, the incidence rate of BC is higher among young individuals. It has been reported that the average age of BC patients in East Asia is more than 10 years younger than that in Europe and the United States. Patients younger than 35 years old account for 8–10% of the total BC population. It is estimated that in China alone, the number of young patients with BC is more than 50,000 (2). The younger trend of BC incidence needs to be considered and valued.

The relationship between age and the outcome of neoadjuvant chemotherapy (NAC) remains controversial. Although compared with elderly patients, younger BC patients tend to have larger tumor diameters and more aggressive tumors (3–5), some studies have shown that the rate of pathological complete response (pCR) after NAC in younger BC patients is higher than that in elderly women. For example, the GeparTrio study reported that the pCR rate of patients younger than 40 years old was 31%, while in patients older than 40 years old, the pCR rate was 18% (6). Similar conclusions were confirmed by several studies (7–9). However, some studies indicate that the effect of age on the outcome of NAC varies with BC molecular subtype (10–13). In hormone receptor (HR)+/human epidermal growth factor receptor 2 (HER2)- and triple-negative breast cancer (TNBC) subtypes, young patients are more likely to reach pCR, while there is no such difference in other subtypes of BC. Due to a lack of direct evidence of the relationship between age and the efficacy of NAC, neither the National Comprehensive Cancer Network (NCCN) nor the European Society of Medical Oncology (ESMO) guidelines recommend age as the only basis for BC patients to receive NAC.

One of the main purposes of NAC is to reduce tumor stage so that patients can obtain the opportunity to receive breast-conserving surgery (BCS). In a prospective study, the NAC rate and clinical complete remission rate increased significantly from 2006 to 2016, but the incidence of BCS in young BC patients did not increase (14). This study suggests that although the proportion of young women eligible for BCS has increased after NAC, patients will still choose mastectomy due to social and psychological factors.

At present, because of the high incidence rate of BC and the trend of youth, BC in young women has become an important public health problem. The existing research is contradictory to whether young BC patients have unique clinicopathological characteristics and survival outcomes. High-quality clinical research evidence is urgently needed to guide the treatment of young patients. As an ideal treatment for young patients, BCS can greatly reduce the physical and psychological trauma of patients. There is a lack of relevant statistical research on the current trend of surgical treatment and the acceptance rate of BCS for young BC patients in China. In this multicenter real-world study, we sought to analyze the outcome of NAC as well as the current status and trend of surgical decision-making after NAC in young BC patients.

## 2. Materials and methods

### 2.1. Study design

**Figure 1:**
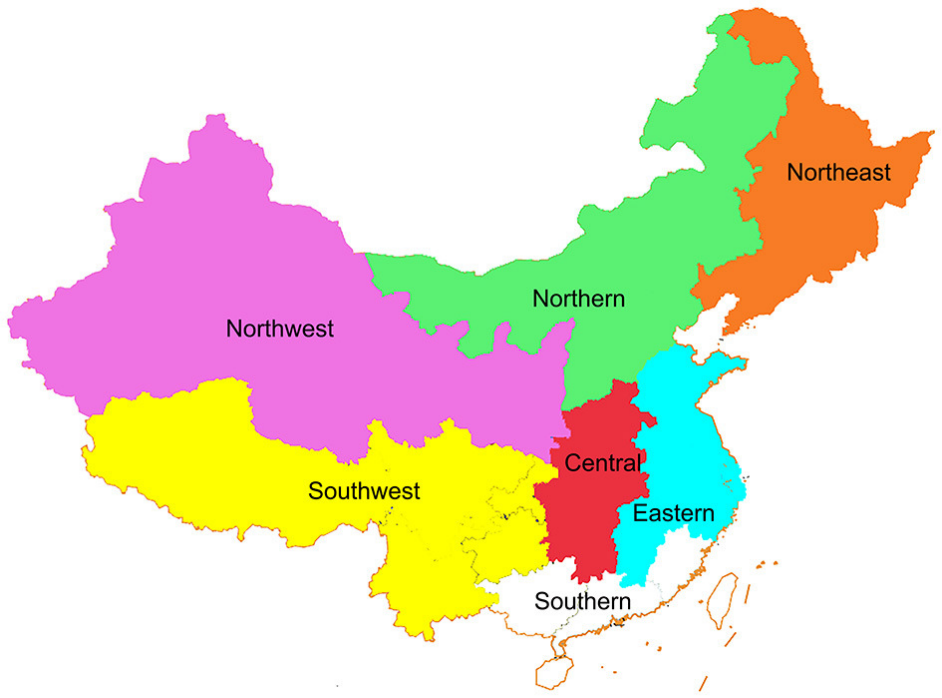
Geographical distribution of hospitals included in the CSBrS-012 study. Colored areas are included in our study, while white areas are not.

This study is a multicenter real-world study. The Chinese Society of Breast Surgery (CSBrS) conducted retrospective data collection in 20 hospitals in different regions of China (CSBrS-012 study). The 20 hospitals are located in central, northern, eastern, northwest, northeast, and southwest China (Figure 1). All hospitals are required to collect the information of BC patients who meet the inclusion and exclusion criteria from January 2010 to December 2020 and report the general characteristics, disease information, treatment plans and outcomes of patients according to the case report form (CRF) designed by CSBrS. The study was carried out in accordance with the Helsinki Declaration and was approved by the Ethical Review Committee of the First Affiliated Hospital of Xi'an Jiaotong University. Since this is a retrospective study and all data analysis was conducted anonymously, the Ethical Review Committee exempted the informed consent of patients.

### 2.2. Patient inclusion

**Figure 2:**
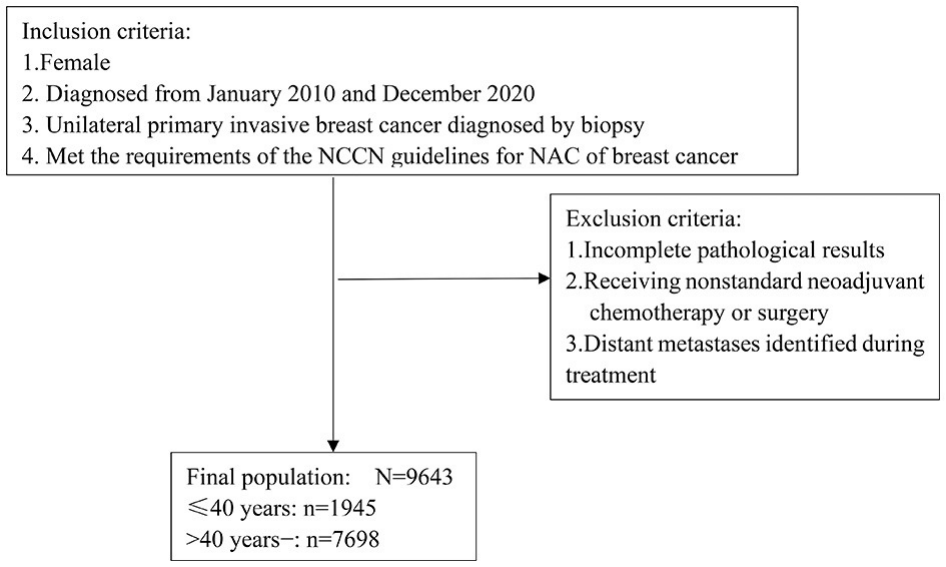
Flow chart of patient inclusion and exclusion. NAC, neoadjuvant chemotherapy; NCCN, National Comprehensive Cancer Network.

CSBrS-012 set unified criteria for the inclusion and exclusion of subjects for all hospitals to reduce the selection bias of subjects. The enrolled population included females who were pathologically diagnosed with cT1-4N0-3 unilateral primary invasive BC from January 2010 to December 2020 and who met the indications for NAC in the NCCN Breast Cancer Guidelines. Patients who had incomplete pathological results, nonstandard neoadjuvant chemotherapy or surgery treatment, or distant metastasis found during treatment were excluded. The detailed screening criteria for patients are shown in Figure 2. All the treatment plans of the included patients were in line with the NCCN Breast Cancer Clinical Practice Guidelines and the Chinese Society of Clinical Oncology (CSCO) Breast Cancer Diagnosis and Treatment Guidelines.

### 2.3. Factor collection and end points

The patient data collected included age, chemotherapy scheme, chemotherapy cycle, clinical stage, Ki-67 index, molecular typing, pathological results, etc. The histological type was in accordance with the World Health Organization (WHO) classification criteria for breast tumors. We determined the molecular type for BC patients according to the St. Gallen Guidelines guidelines (15): (I) HR-, HER2+; (II) HR+, HER2+; (III) TNBC: HR-,HER2-; (IV) Luminal A: HR+, HER2-, Ki67 <20%; (V) Luminal B: HR+, HER2-,Ki67≥20%. The clinical stage of the patient was based on the Union for International Cancer Control tumor-node-metastasis (UICC TNM) system. pCR after NAC was defined as no residual invasive tumor found in the primary breast lesions according to the postoperative pathological results, namely, bpCR (ypT0/is). The clinical and pathological information of patients was collected from the electronic medical system by two independent researchers and examined by a third researcher, which ensured the accuracy of the data and reduced the information bias of the study. CSBrS randomly selected 5% of the forms from each hospital for quality inspection.

### 2.4. Statistical analysis

We performed the chi-square test to compare the differences between groups. Descriptive statistics were used to explain the distribution and characteristics of the study population and are displayed as figures, averages and percentages. Logistic regression was used to identify the predictors related to pCR. In order to reduce confounding bias, we conducted a hierarchical analysis of the variable factors that may affect the outcome. The *P* values were double tailed, and *P* < 0.05 was considered statistically significant. All statistical analyses were performed using IBM SPSS Statistics software version 22.0 (IBM Corporation, Armonk, NY, USA).

## 3. Results

### 3.1. Patient and tumor characteristics

A total of 9,643 eligible patients were included in this study, 1,945 (20.2%) of whom were ≤40 years old (Table 1). In the young patient group, the average age of diagnosis was 34.5 ± 4.3 years old. Clinical stage II and III patients accounted for 1,275 (65.6%) and 609 (31.3%) respectively. In patients aged ≤40 years, the most common histological subtype was luminal B (733, 37.7%), followed by HR+/HER2+ (424, 21.8%) and TNBC (363, 18.7%). Most patients received chemotherapy based on taxane and/or anthracyclines during NAC. Compared with women over 40 years old, young women tend to have a higher tumor stage, larger tumor diameter, more metastatic lymph nodes and higher expression of the Ki-67 index. At the same time, the proportion of Luminal B and TNBC molecular subtypes in young patients was higher than that in >40-year-old patients (*P* < 0.001).

**Table 1:** Patient characteristics in the ≤40 year group and >40 year group.

**Patient characteristic**	**Age group**		***P***
	≤**40 years**	>**40 years**	
*N*	1,945 (20.2%)	7,698 (79.8%)	
Age (years, mean ± SD)	34.5 ± 4.3	52.3 ± 7.0	<0.001
Clinical stage			0.803
I	61 (3.1%)	262 (3.4%)	
II	1,275 (65.6%)	5,060 (65.7%)	
III	609 (31.3%)	2,376 (30.9%)	
Clinical T stage			<0.001
cT1	212 (10.9%)	974 (12.7%)	
cT2	1,234 (63.4%)	5,148 (66.9%)	
cT3	386 (19.8%)	1,127 (14.6%)	
cT4	113 (5.8%)	449 (5.8%)	
Clinical N stage			0.013
cN0	560 (28.8%)	2,126 (27.6%)	
cN1	1,053 (54.1%)	4,011 (52.1%)	
cN2	200 (10.3%)	978 (12.7%)	
cN3	132 (6.8%)	583 (7.6%)	
Histological subtype			0.041
Ductal	1,760 (90.5%)	6,836 (88.8%)	
Lobular	18 (0.9%)	133 (1.7%)	
Mixed	19 (1.0%)	77 (1.0%)	
Other	148 (7.6%)	652 (8.5%)	
Molecular subtype			<0.001
HR-/HER2+	261 (13.4%)	1,311 (17.0%)	
HR+/HER2+	424 (21.8%)	1,734 (22.5%)	
TNBC	363 (18.7%)	1,264 (16.4%)	
Luminal A (HR+/HER2-, Ki67 < 20%)	164 (8.4%)	740 (9.6%)	
Luminal B (HR+/HER2-, Ki67≥20%)	733 (37.7%)	2,649 (34.4%)	
Neoadjuvant chemotherapy plan			<0.001
TAC/AC-T/TA	1,324 (68.1%)	4,949 (64.5%)	
TC/TX/TP/AC	116 (6.0%)	599 (7.8%)	
AC-TH/TCbH	193 (9.9%)	907 (11.8%)	
TCbHP/THP/AC-THP	98 (5.0%)	303 (3.9%)	
Other	212 (10.9%)	919 (12.0%)	
Chemotherapy cycle			0.125
4	297 (15.3%)	1,241 (16.1%)	
6/8	1,330 (68.4%)	5,291 (68.7%)	
>8	142 (7.3%)	454 (5.9%)	
Other	176 (9.0%)	712 (9.2%)	
Ki-67, %	46.5 ± 24.0	42.4 ± 22.9	<0.001

HER2, human epidermal growth factor receptor 2; TNBC, triple negative, negative for ER, PR and HER2; T, Docetaxel; H, Herceptin; X, Capecitabine; P, Carboplatin; A, Doxorubicin; C, Cyclophosphamide; Cb, Carboplatin; P, Pertuzumab.

### 3.2. PCR rates in different molecular subtypes

The pCR rate of the total population was 20.6% (1,986/9,643) and 20.3% (395/1,945) in the young group, which was not significantly different from that in the >40-year-old patients (Table 2). In different molecular subtypes of young patients, the incidence of pCR was higher in TNBC and HR-/HER2+, which was 31.1% (113/363) and 26.1% (68/261), respectively. The difference in the pCR rate among different molecular subtypes in the ≤40-year-old group and >40-year-old group was shown in Table 2. In Luminal B tumor, the pCR rate in ≤40-year-old patients was higher than that in >40-year-old patients, while in the HR-/HER2+ subtype, the >40-year-old group was more likely to achieve pCR (*P* < 0.05). The pCR rates in the HR+/HER2+, TNBC and Luminal A subtype did not differ between age groups.

**Table 2:** The pathologic complete response (pCR) rates in different molecular subtypes of breast cancer patients.

**Molecular subtype**	**Age group**			***P***
	**Overall cohort**	≤**40 years**	>**40 years**	
	20.6% (1,986/9,643)	20.3% (395/1,945)	20.7% (1,591/7,698)	0.726
HR-/HER2+	32.0% (503/4,572)	26.1% (68/261)	33.2% (435/1,311)	0.024
HR+/HER2+	22.4% (484/2,158)	21.2% (90/424)	22.7% (394/1,734)	0.508
TNBC	30.6% (498/1,627)	31.1% (113/363)	30.5% (385/1,264)	0.807
Luminal A (HR+/HER2-, Ki67 < 20%)	5.8% (52/904)	6.1% (10/164)	5.7% (42/740)	0.834
Luminal B (HR+/HER2-, Ki67≥20%)	13.3% (449/3,382)	15.6% (114/733)	12.6% (335/2,649)	0.040

HR, hormone receptor; HER2, human epidermal growth factor receptor 2; TNBC, triple negative, negative for ER, PR and HER2.

### 3.3. Correlation between pCR and clinical factors

To further discuss the clinical characteristics related to pCR after NAC in young BC patients, we conducted logistic regression to analyze possible predictors (Table 3). Univariate logistic regression analysis showed that patients who had stage II disease, cT1 tumors, ductal carcinoma, TNBC subtype, TCbHP/THP/AC-THP chemotherapy plan, 6/8 chemotherapy cycles and high expression of Ki-67 were more likely to achieve pCR. In multivariate analysis, clinical T stage, molecular subtype, NAC regimen, chemotherapy cycle and Ki-67 index were independently related to pCR.

**Table 3:** Logistic regression analysis of clinical characteristics related to pathologic complete response (pCR) after neoadjuvant chemotherapy in young breast cancer patients.

**Patient characteristic**	**Univariate analysis**			**Multivariate analysis**		
	**OR**	**95% CI**	***P*** **-value**	**OR**	**95% CI**	***P*** **-value**
Clinical stage			<0.001			
I	1					
II	1.701	0.828–3.493	0.148			
III	1.081	0.516–2.246	0.836			
Clinical T stage			<0.001			<0.001
cT1	1			1		
cT2	0.864	0.616–1.212	0.398	0.759	0.532–1.081	0.127
cT3	0.457	0.298–0.701	<0.001	0.387	0.248–0.606	<0.001
cT4	0.459	0.246–0.859	0.015	0.378	0.197–0.722	0.003
Clinical N stage			0.629			
cN0	1					
cN1	0.999	0.776–1.287	0.996			
cN2	0.957	0.640–1.431	0.83			
cN3	0.724	0.435–1.205	0.214			
Histological subtype			0.043			
Ductal	1					
Lobular	0.236	0.031–1.780	0.161			
Mixed	0.472	0.109–2.054	0.317			
Other	1.538	1.053–2.246	0.026			
Molecular subtype			<0.001			<0.001
HR-/HER2+	1			1		
HR+/HER2+	0.765	0.533–1.098	0.146	0.712	0.487–1.040	0.079
TNBC	1.283	0.900–1.829	0.169	1.337	0.871–2.053	0.185
Luminal A (HR+/HER2-, Ki67 < 20%)	0.184	0.092–0.370	<0.001	0.334	0.154–0.722	0.005
Luminal B (HR+/HER2-, Ki67≥20%)	0.523	0.372–0.735	<0.001	0.644	0.427–0.972	0.036
Neoadjuvant chemotherapy plan			<0.001			0.002
TAC/AC-T/TA	1			1		
TC/TX/TP/AC	1.062	0.654–1.725	0.806	1.029	0.620–1.708	0.913
AC-TH/TCbH	1.461	1.022–2.089	0.037	1.456	0.934–2.270	0.098
TCbHP/THP/AC-THP	3.001	1.956–4.604	<0.001	2.900	1.744–4.821	<0.001
Other	1.285	0.903–1.829	0.163	1.344	0.913–1.979	0.134
Chemotherapy cycle			<0.001			<0.001
4	1			1		
6/8	2.51	1.693–3.720	<0.001	2.558	1.699–3.853	<0.001
>8	2.914	1.715–4.953	<0.001	2.900	1.676–5.108	<0.001
Other	1.555	0.894–2.705	0.118	1.547	0.875–2.736	0.133
Ki-67, %	1.015	1.011–1.020	<0.001	1.011	1.005–1.017	<0.001

HER2, human epidermal growth factor receptor 2; TNBC, triple negative, negative for ER, PR and HER2; T, Docetaxel; H, Herceptin; X, Capecitabine; P, Carboplatin; A, Doxorubicin; C, Cyclophosphamide; Cb, Carboplatin; P, Pertuzumab.

### 3.4. Current trend of BCS in young patients

**Figure 3:**
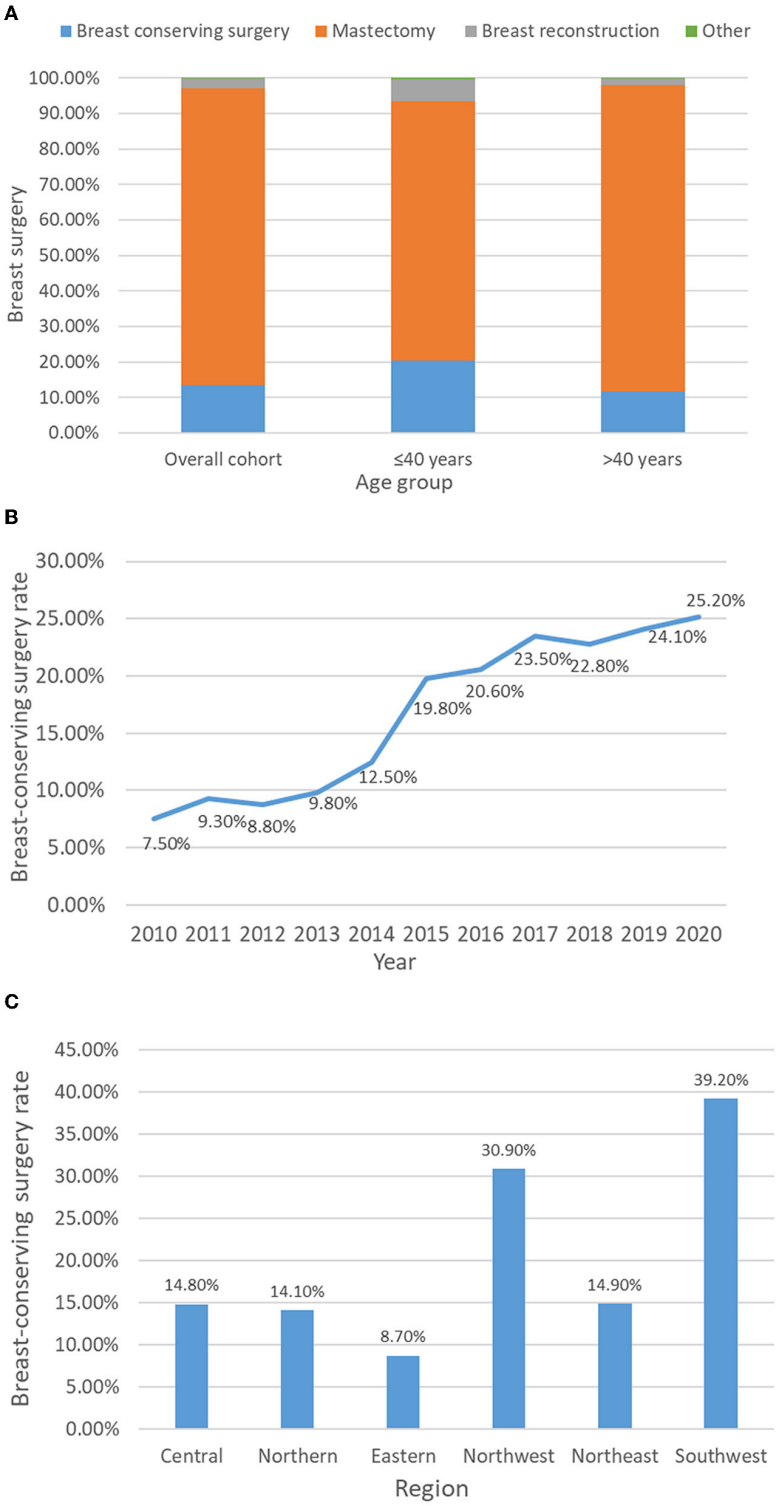
Acceptance rate of breast-conserving surgery after breast cancer neoadjuvant chemotherapy in different age groups **(A)**, years **(B)** and regions of China **(C)**.

After NAC, the percentages of the overall cohort receiving BCS, mastectomy and breast reconstruction surgery were 13.4, 83.6, and 2.8%, respectively. The rates of performing BCS (20.4 vs. 11.7%) and breast reconstruction surgery (6.2 vs. 1.9%) in young patients were higher than those in the >40-year-old group (Figure 3A). Among young patients, the acceptance rate of BCS was on the rise and increased significantly from 7.5% in 2010 to 25.2% in 2020 (Figure 3B). In different regions of China, the implementation rate of BCS varies significantly among young patients. Among them, the BCS implementation rates in the southwestern and northwestern regions were the highest, at 39.2 and 30.9%, respectively, while in the eastern region, only 8.7% of young patients chose BCS after NAC (Figure 3C).

## 4. Discussion

In this study, we conducted a retrospective analysis of 9,643 patients who received NAC from January 2010 to December 2020. Research on population characteristics showed that young patients tend to have a higher tumor stage and a higher proportion of Luminal B and TNBC tumors. There was no statistically significant difference in the pCR rate between ≤40-year-old patients and >40-year-old patients. However, subgroup analysis suggested that the ≤40-year-old group had a higher pCR rate in Luminal B tumor, while the >40-year-old group was more likely to obtain pCR in the HR-/HER2+ subtype. Multivariate logistic regression indicated that clinical T stage, molecular subtype, NAC regimen, chemotherapy cycle and Ki-67 index were independently related to pCR in young patients. The implementation rate of BCS and breast reconstruction surgery was higher in young patients and tended to increase over time. In different regions of China, there are great differences in the choice of surgical treatment after NAC among young patients. To the best of our knowledge, this is one of the largest cohorts of young BC patients receiving NAC and one of the most comprehensive reports on the acceptance of BCS for young BC patients after NAC in China.

Compared with older women, young BC patients have a higher risk of recurrence and a poorer prognosis (16). One of the possible reasons is the higher tumor stage when patients are diagnosed (17, 18), which is consistent with our results. According to the CSCO guideline, women younger than 40 years old are classified as low-risk groups for BC, and it is not recommended to conduct regular BC-related screening for young women, while mammography and ultrasound screening since the age of 40 have greatly improved the detection rate of BC in the elderly population (19). Young women tend to pay less attention to health than elderly women, and a lack of health awareness of BC is also one of the reasons for delays in seeking medical advice. Especially for the changes in breast appearance during pregnancy, puerperium and lactation, young women tend to consider that these are normal physiological changes and ignore the possibility of BC (20). Young BC tumors show different biological behaviors. According to previous studies, the proportion of TNBC in young patients is relatively higher (21). In our study, 18.7% of young patients had the TNBC subtype, and 37.7% were Luminal B tumor, both of which were higher than those in the >40-year-old group. TNBC and Luminal B are the molecular subtypes with the worst prognosis in BC. BRCA1/2 gene mutation may explain the high proportion of TNBC tumor in young patients (22). In addition, even in young patients with HR+, mutations of the PIK3CA gene may lead to resistance to endocrine therapy (23).

Whether age affects the outcome of chemotherapy is controversial. Some researchers believe that young patients are more likely to achieve pCR (8, 13). In a pooled analysis of eight prospectively randomized controlled trials, it was shown that the pCR rate in <40-year-old patients was higher than that in 40–49-year-old and ≥50-year-old patients (20.9 vs. 17.7 vs. 13.7%; *p* < 0.001), and this difference was limited to the HR+/HER2- and TNBC subtypes (8). Verdial et al. believed that the pCR rate of TNBC tumor in young patients was indeed higher, but age did not affect the overall pCR rate (12). Consistently, the above studies all indicated that the influence of age on NAC outcome may vary according to the molecular subtype of BC. Our research data showed that the relationship between age and the pCR rate was not significant. Only in the Luminal B subtype did young patients have a higher pCR rate (15.6 vs. 12.6%, *P* = 0.04). One of the possible reasons for the difference in research results is the definition of outcomes. We define pCR as ypT0/is, while some researchers accept it as ypT0, ypN0 or ypT0/is, ypN0, which are stricter than the definition used in our study. Different outcome criteria will lead to different research conclusions. In addition, the population we studied was Asian. Race affects the outcome of neoadjuvant chemotherapy, which may be related to biological differences in chemosensitivity and socioeconomic factors (24).

Since the European Milan trial and National Surgical Adjuvant Breast and Bowel Project (NSABP) data showed that there was no significant difference in disease-free survival and overall survival between BCS combined with radiotherapy and mastectomy, the long-term safety of BCS has been confirmed (25, 26). In recent years, a study from the Netherlands Cancer Registry suggested that the 10-year survival of patients receiving BCS combined with radiotherapy was better than that of patients receiving mastectomy, which was conducive to the enhancement of local treatment (27). Moreover, BCS has a better cosmetic effect, reduces the psychological burden of patients, and greatly improves the quality of life of young patients (28). Therefore, BCS has become an ideal treatment for early BC. The acceptance rate of BCS varies greatly in different countries and regions. According to data from the Surveillance, Epidemiology, and End Results (SEER) database, the implementation rate of BCS in early BC in the United States is 55–60%, and this proportion is increasing yearly (29). Data from more than 20 BC centers in Europe showed that the acceptance rate of BCS is 75–80% (30). However, BCS is not popular in China. The BCS rate in China was 1.29% in 1999 and 11.57% in 2008. Even in economically developed areas such as Beijing and Shanghai, the BCS rate was only 24.3% in 2008 (31, 32). Most Chinese female patients chose mastectomy after NAC (83.6%), while among young women, the proportion was still as high as 73.1%. Increasing the possibility of breast preservation is not the main purpose of Chinese women receiving NAC.

The acceptance of BCS is affected by many factors, and socioeconomic factors are one of the leading factors (33). Choosing BCS means an extra cost of breast radiotherapy, so the acceptance rate of BCS among low-income groups and low medical insurance groups is relatively low (34, 35). The BCS rate is also affected by the level of regional economic development, which is also confirmed by our research. It is more convenient to carry out BCS in economically developed areas where high-quality medical resources are concentrated. In some areas with poor medical conditions, patients have to give up the choice of BCS due to the lack of radiotherapy equipment and professional radiotherapy doctors. Jeong et al. showed that the proportion of young women eligible for BCS increased significantly after NAC, but a considerable number of patients were still undergoing mastectomy (14). This prospective clinical study indicated that patients' personal preferences also played an important role in surgical decision-making. Concerns about tumor recurrence and the side effects of radiotherapy make patients prefer to choose total mastectomy, which is more obvious among women with a low education level (36, 37). Doctor–patient communication on the safety of BCS will also affect the acceptance rate of BCS, including the communication skills of doctors, the frequency of communication and the cognitive ability of patients. In future research, the influence of the social economy and tumor psychology on patients' surgical treatment decision-making should be emphasized, especially for young cancer patients.

As a real-world study, there are inevitably some deficiencies. First, most of the 20 hospitals included in the CSBrS Alliance are first-class hospitals, which have relatively high-quality medical conditions in the region, so there may be some bias in the current research. In addition, we lack relevant data from patients in southern China, which may affect the presentation of the overall results. However, the 20 hospitals included in CSBrS have covered regions with different economic development levels in China, and it is the largest and most comprehensive retrospective cohort study on NAC of BC in China thus far. Therefore, we believe that our study can reflect the overall level of diagnosis and treatment of BC in China to a certain extent. We will further include more subcenter hospital data to make our research more representative. Second, doctors are subjective in assessing whether patients have BCS qualifications, which may affect the choice of surgical treatment. Third, there were pCR-related factors in young patients who were not included in our analysis, such as BRCA mutation status, family history, and histological grade. Our research conclusion needs to be verified by a larger population and a longer follow-up time. We will follow up with the latest statistics to conduct validation-related supplementary research in our further study.

## 5. Conclusion

Young BC patients tend to have a higher tumor stage and a higher proportion of Luminal B and TNBC tumors than elderly patients. Age does not affect the overall pCR rate, but the Luminal B tumor was more likely to obtain pCR in young patients. In different regions of China, there are great differences in the choice of surgical treatment after NAC. The BCS rate of young Chinese BC patients after NAC has increased over time but is still at a low level. In the individualized precise treatment of BC, young patients need special attention due to their unique clinical characteristics. NAC may be especially recommended for young BC patients with Luminal B subtype. In future research, the influence of the social economy and tumor psychology on patients' surgical treatment decision-making should be emphasized, especially for young cancer patients.

## Data availability statement

The raw data supporting the conclusions of this article will be made available by the authors, without undue reservation.

## Ethics statement

The studies involving human participants were reviewed and approved by the Ethical Review Committee of the First Affiliated Hospital of Xi'an Jiaotong University. Written informed consent for participation was not required for this study in accordance with the national legislation and the institutional requirements.

## Author contributions

JH, ZF, and HZ designed the research, the corresponding authors who revised the manuscript, and supervised the completion of the study. YL and HC analyzed the data. YL, HC, and HZ drafted the manuscript. All authors contributed to the article and approved the submitted version.
